# Dry Anophthalmic Socket Syndrome—A Narrative Review

**DOI:** 10.3390/jcm14248814

**Published:** 2025-12-12

**Authors:** Daniele Lorenzano, Alberto Chierigo, Alessandra Claudia Modugno

**Affiliations:** 1 Oculoplastic and Orbital Service, Department of Ophthalmology, University Hospital Southampton NHS Foundation Trust, Southampton SO16 6YD, UK; 2 Ocularistica Italiana, 00192 Rome, Italy

**Keywords:** DASS, dry anophthalmic socket syndrome, dry anophthalmic socket, anophthalmic socket, dry socket

## Abstract

Dry Anophthalmic Socket Syndrome (DASS) is a multifactorial condition that affects roughly half of all prosthetic eye wearers and remains frequently underrecognized. It is characterised by symptoms such as dryness, discomfort, discharge, and inflammation of the socket surface. Diagnostic criteria include validated symptom questionnaires (e.g., OSDI, DEQ-5, SANDE) and at least one clinical sign such as conjunctival staining, blepharitis, or reduced tear meniscus height. This review describes the anatomical, cellular, and molecular changes associated with DASS. Meibomian gland dysfunction is common, with a significant reduction in gland density and structure. Goblet cell density is also often decreased, particularly in the tarsal and bulbar conjunctiva, although findings may be affected by topical treatments. Increased conjunctival inflammation—evidenced by immune cell infiltration and elevated markers such as MMP-9 and ICAM-1—is frequently observed, particularly in the posterior socket lining. Oxidative stress, mediated by dysregulated NOX4, KEAP1, and NRF2 expression, appears to play a contributory role. Additional factors influencing DASS include eyelid malpositions such as entropion and ectropion, prosthesis smoothness and amount of tear film production. Poor hygiene practices and environmental factors may exacerbate symptoms. Given its multifactorial aetiology, DASS requires a complex management strategy targeting inflammation, tear film instability, mechanical irritation, eyelid position and patient education. Increased awareness, standardised diagnostics, and evidence-based care protocols are critical to improving outcomes for prosthetic eye wearers.

## 1. Introduction 

It has long been known that artificial eye wearers may experience dryness and discharge caused by changes in the socket lining [1,2]. This phenomenon has more recently gained the formal definition of Dry Anophthalmic Socket Syndrome (DASS) and, despite being frequently underrecognized, affects roughly half of prosthetic eye wearers [3]. The importance of DASS is further underlined by the fact that frequency of lubrication was the most predictive factor in overall prosthesis comfort [4].

In this narrative review we summarise the existing literature on DASS, providing anatomical, molecular and clinical findings, highlighting both established and emerging evidence.

Like other forms of dry eye, DASS can be defined as a multifactorial disease of the socket leading to discomfort and characterised by loss of tear film homeostasis, which stems from tear film instability, meibomian gland dysfunction, and subclinical inflammation [3,4,5,6]. However, the presence of an artificial eye is a key feature that differentiates DASS from other forms of dry eye disease, as this further complicates the pathophysiology and treatment.

DASS can only be diagnosed when a patient with an anophthalmic socket fulfils the following criteria (Table 1) [3]:

- Subjective symptoms at the anophthalmic socket, evaluated with standardised measurements: OSDI ≥ 13 or DEQ-5 ≥ 6 or SANDE ≥ 13;
- At least one of the four following clinical abnormalities: significant conjunctival inflammation resulting in conjunctival staining, reduced tear meniscus height, anterior blepharitis, posterior blepharitis.

Of note, it is sufficient to use a single questionnaire for each patient, but separate results should be obtained for the socket and the fellow eye. In addition to this, it might be more appropriate to use questionnaires that do not include items on visual function (such as the OSDI) when dealing with anophthalmic patients.

The use of the Schirmer test is not considered to be evidence-based and can therefore be omitted in clinical practice [3].

## 2. Materials and Methods

A comprehensive literature search was performed on three bibliographic databases—MEDLINE (via OVID), EMBASE (via OVID) and Web of Science—searching for relevant literature. Articles published up to the 28 July 2025 were included.

A comprehensive search strategy was developed using medical subject headings (MeSH) and equivalent free-text words in order to capture all the studies addressing the research question. Terminology relating to “anophthalmic socket”, “dry socket”, “ocular prosthesis”, “tear film”, “socket discomfort”, “socket inflammation”, and “dry anophthalmic socket syndrome” were included. Truncation symbols, wildcards, and proximity operators were also utilised. Key terms were combined using Boolean operators. The search strategy was initially conducted on MEDLINE and was then adapted for the other databases, adjusting for variations in syntax.

Due to the paucity of literature in this field, eligible articles included original research studies, clinical trials, case series, and relevant review articles. Studies were selected based on their relevance to the clinical presentation, underlying mechanisms, diagnostic approaches, and treatment options for DASS. Reference lists of included articles were also screened to identify additional pertinent literature. Only studies published in English were included. We excluded all studies that were not in English or that did not specifically address dry anophthalmic socket syndrome.

### Data Extraction

When available, the following data were extracted from each eligible study: article information (authors, study design, year), objectives, study cohort, sample size, patients’ characteristics, clinical manifestations, involved organs, follow-up, risk factors, and outcomes.

Findings from the selected literature were synthesised qualitatively to provide a cohesive summary of current knowledge on the topic.

## 3. Results

### 3.1. Meibomian Glands

**Figure 1:**
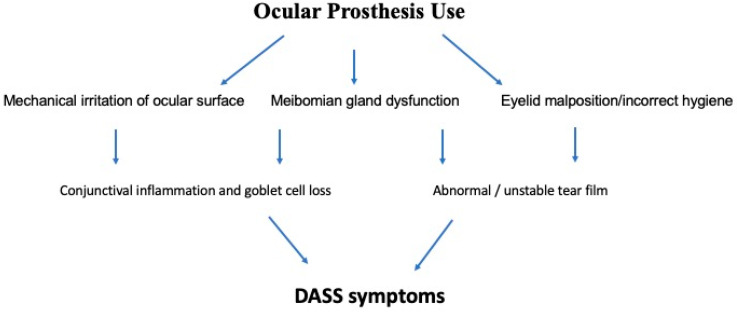
Schematic diagram of the pathophysiology of dry anophthalmic socket syndrome (DASS).

Multiple factors conspire to produce inflammation of the socket lining, with chronic mechanical irritation and tear film instability being two key offending elements (Figure 1).

Continuous contact and friction against the prosthesis have been shown to produce abnormalities in multiple anatomical structures, leading to disruption in tear film homeostasis.

Among the anatomical structures involved in such process, meibomian glands have been demonstrated to undergo changes in number and structure in anophthalmic sockets compared to fellow eyes, ultimately leading to meibomian gland dysfunction (MGD).

Meibography of the upper and lower lids of artificial eye wearers revealed a significant drop in gland numbers in the eyelids of a prosthetic eye compared to the normal eyelids, with median total meibography scores of 3 and 1, respectively, for anophthalmic and fellow eyes (*p* < 0.001) [7]. Of note, this study did not find any difference in upper and lower lid scores but did report more compromised glands after longstanding (>10 years) wear [7]. This study was limited by a small sample size and by failure to include data on prosthesis use or care, as well as prosthesis size or presence of pegs.

Using the same method, Volpe et al. found also found MGD score to be higher in anophthalmic sockets than in healthy fellow eyes [5]. Their study also suffered from a relatively small sample size.

These results were confirmed by a study conducted by Rokohl et al., who performed confocal microscopy of the central part of lower eyelids of anophthalmic socket and fellow healthy eyes [6]. Compared to fellow eyes, anophthalmic sockets had a lower acinar unit density (75.14 ± 18.92 vs. 57.99 ± 9.74 units/mm^2^, respectively) (*p* = 0.003), more inhomogeneous appearance of the acinar unit interstice (grade 1.67 ± 0.62 vs. 2.60 ± 0.83, respectively) (*p* = 0.018) and more inhomogeneous appearance of the acinar unit walls (grade 2.07 ± 0.88 vs. 2.93 ± 0.70, respectively) (*p* = 0.015) [6]. No differences were found in gland size, i.e., acinar unit diameter and area, or in meibum reflectivity. This study was limited by a relatively small sample size and by inclusion of patients with eyelid malpositions, variable prosthesis hygiene and exposure to predisposing environmental factors [6].

### 3.2. Ocular Surface Pathophysiology

#### 3.2.1. Goblet Cells

A lower goblet cell density was seen in anophthalmic sockets as compared to healthy fellow eyes, and this was significant for all parts of the socket lining, with 6.7 ± 10.8 and 16.1 ± 21.8 (*p* = 0.028) cells/5 mm^2^ for bulbar, 7.8 ± 14.1 and 16.3 ± 17.4 (*p* = 0.015) for upper tarsal and 8.9 ± 15.6 and 26.1 ± 29.8 (*p* = 0.002) for lower tarsal conjunctiva, respectively, for anophthalmic sockets and fellow eyes [8]. Notably, this study was limited by heterogeneity in topical medication use, as it included patients on antibiotic-steroid mixture, artificial tears, antihistamine and antibiotics. Moreover, it did not investigate possible presence of benzalkonium chloride in the topical formulations used, and this preservative is known to have a cytotoxic effect on conjunctival goblet cells [9,10]. This might limit the strength of these results. Also, Chang et al. did not find a significant reduction in goblet cell numbers after performing impression cytology in anophthalmic patients with giant papillary conjunctivitis [11]. However, these results are limited by a small sample size (12 patients).

The observed differences in goblet cell populations found by the two studies above could be explained by several factors. One of these could be a different patient population, with Kim et al. having only one patient with papillary conjunctivitis, while Chang et al. had the whole cohort consisting of such patients. Another possible cause of this observed difference could be a small sample size, with Kim et al. and Chang et al., respectively, including 40 and 12 patients. Moreover, patients studied by Kim et al. might have used drops with preservatives that are toxic to goblet cells, as stated above.

Changes in the conjunctival goblet cells are not limited to their numbers on impression cytology, as demonstrated by recent studies on the expression of certain molecules [5,12].

One of these molecules is the mucin MUC5AC, which is secreted by conjunctival goblet cells onto the ocular surface and is responsible for the rheological properties of mucus [13]. This mucin has been shown to be a marker of goblet cell numbers and function, and its expression decreases in dry eye disease [14,15].

As for the anophthalmic socket, Volpe et al. reported a significant increase in the expression of MUC5AC in the posterior conjunctiva of the socket when compared to healthy controls (log2 fold change 6.60, *p* < 0.001) [5]. Interestingly, this is the opposite of what has been demonstrated for dry eyes. The authors speculated that the increased MUC5AC expression on the posterior socket surface might represent a localised compensatory mechanism to prosthesis contact, which is more prominent in the bulbar conjunctiva [5]. The opposite happened for superior and inferior tarsal conjunctiva, where this mucin was less expressed, but this difference was not statistically significant [5]. This decrease was speculated to arise from a more dynamic relationship with the anterior prosthesis surface. Alternatively, continuous tarsal rubbing might have affected the compensatory increase in MUC5AC expression [5].

#### 3.2.2. Conjunctival Epithelium—Impression Cytology Studies

Another structure that is considerably altered in the anophthalmic patient is the conjunctival epithelium. The study by Kim et al. showed that the socket epithelial cells undergo squamous metaplasia and increase in the nucleus-to-cytoplasm ratio, and was found at the upper tarsal, bulbar, and lower tarsal conjunctiva (*p* < 0.001, *p* = 0.001, *p* < 0.001, respectively) [8]. This was not correlated to total wearing time, frequency of polishing or frequency of cleaning solution or eyedrops. Interestingly, prosthesis cleaning more than once a day was associated with more changes only in the upper tarsal epithelium, and increased inflammation was correlated to less epithelial abnormalities in the lower tarsal conjunctiva. However, the authors speculated that this might represent a purely statistical, rather than clinically relevant, finding [8]. In addition to this, the same considerations cited above can be made for the use of different topical drugs and possible preservatives, which might have affected the conjunctival epithelium as well.

This data is confirmed by Chang et al., who reported a statistically significant increase the presence of a honeycomb pattern in the upper and lower tarsal conjunctiva of giant papillary conjunctivitis specimens [11]. On the other hand, the same did not seem to occur in the bulbar conjunctiva [11].

#### 3.2.3. Conjunctival Inflammation

**Figure 2:**
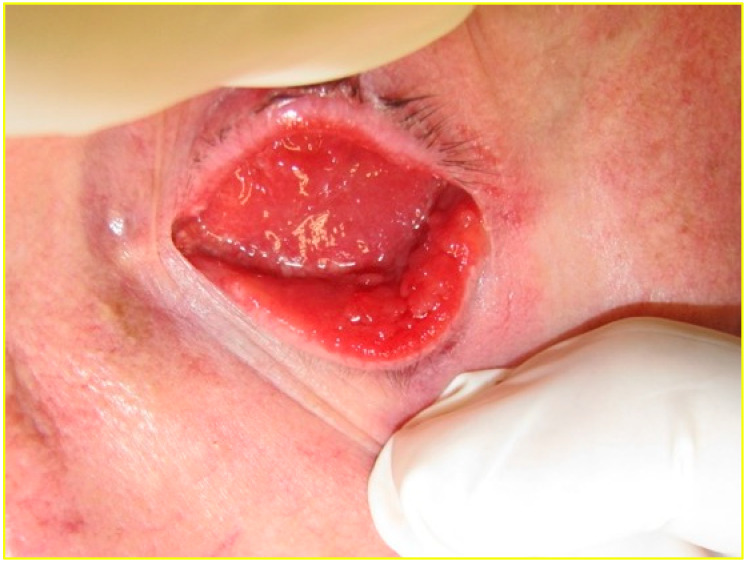
Inflamed socket with giant papillary conjunctivitis of the bulbar and tarsal conjunctiva.

Several studies have reported that the conjunctiva of a socket is more inflamed than that of the fellow eye [3,5,6,8,11,12,16,17,18]. This inflammation often manifests macroscopically as conjunctival papillae, redness, chemosis and discharge (Figure 2) [17].

The exact mechanism underlying the formation of papillae is unknown, but it is hypothesised that a major cause is a mechanical insult to the epithelium and an immunologic response to deposits on the prosthesis [19].

In an impression cytology study on anophthalmic patients with giant papillary conjunctivitis (GPC), conjunctival inflammation was present in the whole socket lining, i.e., on the upper tarsal, bulbar and lower tarsal conjunctiva [11]. The inflammatory infiltrate was composed predominantly by a variable number of polymorphonuclear leukocytes, and mast cells and eosinophils were identified only in a few patients [11].

Bozkurt et al. used immunohistochemical analysis on GPC patients and found a different infiltrate frequency and composition [20]. Eosinophils were found in more than half of all GPC specimens and were more frequently seen in GPC patients as compared to controls. Their number was also higher than in fellow eyes (*p* = 0.025). Their location did not differ from healthy specimens, as they remained in the substantia propria (SP) [20]. Based on these results, they concluded that prosthesis-related GPC might be an allergic disease.

Similarly to the study by Chang et al., mast cells were found in only a few specimens. They permeated into the conjunctival epithelium, while in fellow eyes they remained confined to the SP, and degranulation was prominent in all GPC specimens [20].

The same paper also reported an increase in the number of CD8+ lymphocytes compared with fellow eyes (*p* = 0.012), and these were present in both the epithelium and SP. CD4+ cells were also increased, but less so, and were located mainly in the SP [20].

The expression of inflammatory markers such as matrix metalloproteinases 9 (MMP-9) and intercellular adhesion molecule 1 (ICAM-1) in DASS has also been investigated [5].

MMP-9 is an enzyme that breaks down denatured collagen, native collagens type IV, V, and VII, and elastin. Through this action, it plays a role in inflammation of the ocular surface, and its activity and expression were proven to be significantly increased in patients’ ocular surface disease [21,22]. MMP-9 activity and expression are correlated strongly with dry eye symptoms, and an increased expression of this enzyme’s mRNA was found in the posterior conjunctiva of anophthalmic sockets (*p* < 0.001) [5,21,22]. Interestingly, the upper tarsal conjunctiva showed a downregulation of MMP-9 (*p* < 0.05), indicating a different involvement of anterior and posterior surfaces in DASS [5].

The same differential expression between anterior and posterior surfaces of the anophthalmic socket was found for ICAM-1, an integrin ligand that promotes lymphocyte activation and migration to the ocular surface [5,23]. Again, this molecule’s mRNA was upregulated in the posterior (*p* < 0.001), but not in the anterior socket lining [5].

Notably, the duration of inflammation might be more important than its severity, since no association was seen between conjunctival inflammation severity and DASS symptoms [3]. This likely means that socket inflammation needs to be chronic to produce changes in the ocular surface and, consequently, in the quality of life of artificial eye wearers [3].

#### 3.2.4. Oxidative Stress

Oxidative stress is involved in the pathogenesis of several eye diseases, including several forms of ocular surface inflammation such as dry eye, atopic keratoconjunctivitis and corneal inflammation [24]. A study investigated the expression of three key mediators in the production of and response to reactive oxygen species (ROS) in DASS [5]. These were NOX4, an enzyme that produces ROS, and KEAP1 and NRF2, two molecules involved in the antioxidant response.

NOX4 is an enzyme that belongs to the NADPH oxidases family, a group of enzymes that generate ROS [25]. NOX4 appears to be upregulated under hypoxic and inflammatory states, including dry eye disease, through direct and indirect pathways which involve several transcription factors, such as the hypoxia-inducible transcription factor HIF-1α and the tumour necrosis factor alpha (TNF-α) [26,27,28]. 

NRF2 is a transcription factor that can upregulate the expression of several genes involved in the antioxidant response. Under basal conditions, it is sequestered in the cytoplasm by KEAP1 but, after exposure to high ROS levels, NRF2 moves into the nucleus to drive expression of genes involved in the antioxidant response [29,30,31,32].

Expression of NOX4, NRF2 and KEAP1 was found to be increased in the posterior conjunctiva of anophthalmic sockets (*p* < 0.001, *p* < 0.05 and *p* < 0.001, respectively) [5]. Interestingly, NOX4 and KEAP1 were significantly downregulated in the upper tarsal conjunctiva and NOX4 also in the lower tarsal conjunctiva (*p* < 0.001 for all results) [5].

The authors speculated that extended wear of the artificial eye might expose the bulbar conjunctiva to hypoxia, altered tear film renewal, insufficient supply of antioxidants and, ultimately, contribute to conjunctival inflammation [5]. This hypothesis would explain the expression pattern seen in these patients, with upregulation of NOX4, NRF2 and KEAP1 expression in the bulbar conjunctiva. The downregulation of these markers in the tarsal conjunctiva would be explained by blinking, which would constantly renew the tear film and protect the conjunctiva from hypoxia and ROS [5].

### 3.3. Tear Film

Patients with anophthalmic sockets frequently report dry eye-like symptoms, even in the absence of clinical blepharitis, suggesting that tear misdistribution and evaporative deficits play a key role [3,5]. Earlier studies demonstrated how eye removal eliminates corneal sensory input, leading to a reduction in reflex tear secretion. This was shown by lower Schirmer test results in the anophthalmic socket compared to the healthy contralateral eye [2]. However, more recent research seems to challenge this notion, as direct assessment of tear secretion from the lacrimal gland was largely similar between the anophthalmic socket and the fellow eye [33]. This suggests that aqueous tear deficiency may not be the primary driver of DASS symptoms, with tear misdistribution, MGD and evaporative dry eye playing a more critical role [3,7]. Indeed, meibography studies show that meibomian glands are significantly fewer and more irregular in prosthetic eye wearers, reducing the lipid layer of the tear film and increasing evaporative loss [6]. Fourier domain optical coherence tomography (FD-OCT) measurements further support tear film instability in DASS, revealing significantly lower tear meniscus height, depth, and volume in anophthalmic sockets compared to normal eyes [34].

Tear misdistribution describes the abnormal pooling of tears behind the prosthesis, a phenomenon that is more like to occur with glass prosthesis, as they have a hollow back surface [3]. This is speculated to contribute to DASS, as a lower amount of tears would be available to lubricate the anterior socket lining.

Abnormalities in the tear film are also reflected by elevated tear film osmolarity TFO and MMP-9 levels in anophthalmic sockets as compared to healthy fellow eyes [5,12]. Elevated TFO and MMP-9 levels correlate with clinical symptoms and conjunctival inflammation, increasing objective measures for disease severity and treatment response [12].

Consequently, effective management strategies should address both aqueous and lipid tear deficiencies, potentially incorporating punctal occlusion, artificial tears, anti-inflammatory agents, and targeted therapies for MGD [35,36].

### 3.4. Allergy and the Environment

Few papers mentioned allergy, pollution or the environment in the context of DASS.

A classic example of allergy is GPC, which is thought be caused by a reaction to antigens on the artificial eye surface and is a well-known cause of discomfort and discharge in patients with an anophthalmic socket [3,5,37]. The allergic nature of GPC was demonstrated by immunohistochemical analysis of the socket lining, showing higher populations of mast cells, CD4+ lymphocyte, CD8+ lymphocyte and eosinophils in prosthesis-related GPC specimens compared with fellow eyes (*p* = 0.005, 0.074, 0.012 and 0.025, respectively). Eosinophils were also detected more frequently in prosthesis-related specimens than in controls (58.8% and 16.7% of specimens, respectively, *p* = 0.053) [20]. Moreover, a higher number of inflammatory cells expressed eotaxin and interleukin-4 the GPC group (*p* = 0.050 and 0.048, respectively) [20].

Similarly to other dry eye diseases, certain factors can worsen symptoms and should be controlled whenever possible. These include smoke, dry air, draughts, dust, extensive computer work, air conditioning and vapours, which were found to be associated with higher OSDI scores (*p* = 0.044) and to cause increased DASS-related complaints [3]. This is especially true for discharge, which has been demonstrated to increase after stress, lack of sleep, upper respiratory tract infections, and after exposure to pollens, dust, or smoky air [18]. Conversely, holidays and maritime clime were associated with less discharge [18].

### 3.5. Prosthesis Material and Polishing

The type of prosthetic material (PMMA or cryolite glass) is another important factor to consider in DASS, as it can affect tear distribution. Indeed, glass is more hydrophilic than PMMA, and glass eyes have a hollow back surface that might provide a reservoir of tears [3]. It is unclear whether this reservoir protects against excessive socket dryness or contributes to tear maldistribution and pooling of cytokines against the posterior conjunctival surface.

Despite this, no difference was found in terms of DASS-related symptoms, such as eye watering, discharge and crusting, even though cryolite eyes have been associated with increased comfort (*p* < 0.001) [5,38]. Moreover, clinical and molecular signs of inflammation were also found to be similar between the two materials [5]. However, this latter study was partly limited by a small proportion of cryolite glass prosthesis wearers.

Over time, prostheses usually develop small scratches and surface irregularities that can cause mechanical irritation of the conjunctiva. For this reason, artificial eyes need to be regularly checked, polished or renewed by an ocularist, and the type and frequency of maintenance depends by prosthesis material [39].

In general, yearly polishing of PMMA and renewal of cryolite eyes is recommended [40].

### 3.6. Prosthesis Care

An evidence-based standard on how to ideally care for the prosthesis is currently lacking [4]. The literature seems to indicate that the artificial eye should be cleaned once every 1–6 months [3,4,18,41]. Beyond this general number, it is important to note that mistakes in prosthetic hygiene have been shown to exacerbate DASS symptoms [17,18]. Excessive prosthesis cleaning, i.e., cleaning the prosthesis more than once a month, was consistently associated with increased socket inflammation and discharge [3,4,18,42]. Moreover, too frequent washing of the artificial eye was negatively correlated with higher comfort (*β* = 0.16, *p* = 0.043) [4]. This is likely because too frequent cleaning to disrupts the mucous coating and deposits that build up on the surface of the prosthesis, which are thought to normally provide a more wettable surface. Therefore, excessive cleaning increases mechanical stress on the conjunctiva, leading to inflammation [18].

There is insufficient data in the literature to determine if a specific product or technique should be used for prosthesis cleaning. It is reported that patients currently use one of several combinations of soap, water, saline, disinfectant and antiseptic solutions, but no article has specifically compared cleaning methods and found one that is superior [3].

Another important aspect of artificial eye care is hand washing before removal and insertion of the prosthesis. This step seems to be essential for preventing spread of microorganisms from the hands to the socket and, consequently, to avoid increased inflammation and discharge [3,18,41,43].

Table 2 summarises the essential and ancillary diagnostic tools that allow DASS diagnosis.

### 3.7. Eyelids

Lower lid entropion, ectropion and lagophthalmos in artificial eye wearers are reported with variable frequency in the literature. Of the three, lagophthalmos seem to be the most common, followed by entropion and ectropion [3,12,44]. While the detrimental effects of lower lid malpositions on patient comfort and ocular surface health seem evident, special attention has been drawn to lagophthalmos.

Indeed, incomplete lid closure was found in up to 80% of patients in one paper, whereas another article revealed a frequency of only one third [3,44]. This was reportedly related to better ocularist service in the latter group [3]. Moreover, Rokohl et al. found lagophthalmos, but not lower lid entropion, to be highly associated with socket inflammation [3].

To ensure adequate lubrication of the artificial eye and consequently reduce DASS symptoms, it seems therefore sensible to reduce lagophthalmos and to improve lid position whenever possible. This can be achieved with changes in the prosthesis or with surgical procedures.

### 3.8. Treatment

There is currently no specific treatment guideline for DASS. However, as illustrated above, DASS shares many features with other forms of dry eye disease (DED). At the same time, lack of the eye and presence of a prosthesis are two key features that require special attention and are not encountered in classic DED.

By and large, treatment strategies have been borrowed from DED management, with focus on addressing both aqueous and lipid tear deficiencies [35,36].

Reasonable first line management could consist of artificial tears, warm compresses and lid hygiene, along with patient education on prosthesis care and annual eye renewal or polishing.

Cyclosporine A 0.05% has been studied in DASS by Han et al., who showed that this drug improved symptoms and tear meniscus depth after 3 months [45]. However, their study was limited by several factors, such as lack of a control group, small sample size (20 patients), use of a questionnaire that is not validated for DASS, and short duration of follow-up.

Vitamin A, anti-inflammatory agents such as steroids or cyclosporin, targeted therapies for MGD such as intense pulsed light and punctal occlusion might represent second-line strategies. Aside from punctal occlusion, we did not find any study that investigated these treatments for DASS, and these could be the object of future research. Nevertheless, our group has found benefit in incorporating vitamin A-containing ointments into this first-line approach.

As for punctal occlusion, Zamorano-Martín et al. conducted a randomised trial that demonstrated the effectiveness of artificial tears plus lid wipes and of punctal plugs in DASS [36]. Both treatments yielded improvements in Standard Patient Evaluation of Eye Dryness (SPEED) score and tear meniscus height (TMH) at 3 months (*p* < 0.001 for all). Of note, punctal plugs were shown to be slightly superior to conservative management in terms of TMH (*p* = 0.012), which went from 0.44 ± 0.39 to 1.18 ± 0.39 for the former (*p* < 0.001) and from 0.49 ± 0.40 to 0.86 ± 0.21 for the latter (*p* < 0.001) [36].

However, patients in the conservative management group did not receive any warm compresses, and adding this could have increased the effectiveness seen in that group. No difference was seen in terms of SPEED score between the two groups.

Moreover, a Cochrane review has shown that punctum plugs have possible adverse reactions such as epiphora, plug displacement, canaliculitis, pyogenic granuloma and dacryocystitis [46]. All of this might make punctal occlusion more suited for a second-line strategy.

### 3.9. Brief Considerations on Eyelid and Socket Surgical Management in DASS

Discomfort in Dry Anophthalmic Socket Syndrome (DASS) is more pronounced in contracted sockets and reduced when an adequate conjunctival surface is preserved [3].

Surgical intervention on the conjunctival lining can restore eyelid opening and closure, thereby rehabilitating the affected fornix through repositioning of the posterior lamella [47].

Eyelid malpositions are amenable to correction with eyelid surgery. Among these, entropion is particularly symptomatic, as tears become trapped between the eyelashes and the prosthetic surface. The aqueous component of the tear film evaporates, while the lipid fraction deposits as a viscous film on the prosthesis, producing significant discomfort.

When DASS occurs in the context of a contracted socket, conservative measures are often insufficient. In such cases, dermis fat grafting is indicated. This procedure deepens the fornices, mitigates cicatricial contraction, and simultaneously addresses orbital volume deficiency. The volume replacement can be modulated intraoperatively by adjusting the quantity of fat implanted, allowing both functional and aesthetic rehabilitation [48].

Finally, surgery should be considered for any eyelid malposition that is thought to contribute to patient discomfort, socket inflammation, or if it has a negative impact on prosthesis stability (Table 3).

## 4. Discussion and Conclusions

Dry Anophthalmic Socket Syndrome (DASS) is a multifactorial condition affecting approximately half of prosthetic eye wearers, yet it remains frequently underdiagnosed and undertreated. Emerging evidence demonstrates significant anatomical and molecular alterations in the anophthalmic socket compared to healthy fellow eyes, including reduced meibomian gland density, altered goblet cell profiles, conjunctival metaplasia, and chronic low-grade inflammation. Elevated markers of oxidative stress and pro-inflammatory mediators further underline the pathophysiological overlap between DASS and other ocular surface diseases.

Despite its clinical significance, standardised protocols for diagnosis, management, and prosthesis care remain limited. Diagnostic criteria now incorporate both subjective symptoms and objective clinical signs, with emphasis on socket-specific evaluation tools.

Management strategies should be multifaceted, targeting aqueous tear deficiency, lipid layer insufficiency, inflammation, mechanical factors, and patient-specific contributors such as prosthesis material and hygiene habits.

### Limitations of the Current Literature and Future Directions

We found that several studies on DASS did not have a standardised design. We would like to stress that authors should use standardised diagnostic criteria and only include patients that can be formally diagnosed with DASS using the criteria described by Rokohl et al., which are summarised in Table 1 of this manuscript. We do appreciate that these were first published in 2020, and all studies on the health of the socket lining that were published before this year could not use these diagnostic criteria. However, several subsequent studies still failed to use such standardised criteria and simply included patients with good prosthesis fitting but no explicit diagnosis of DASS. This might induce a bias by including healthy patients with an anophthalmic socket that do not have DASS.

Another area of current gap in our knowledge is stratification of DASS severity. It is reasonable to postulate that this condition might have a spectrum of severity, with some patients having only mild signs or symptoms, while others might experience a more severe form of this disease. At the moment, there are no standardised criteria to determine how severe the disease is, and future literature should address this issue.

Small sample size was an additional key limiting factor of all the studies found, with all articles managing to include roughly fewer than 50 patients.

Another limitation found in most studies was inclusion of patients that wore prosthesis made of different materials (PMMA and cryolite glass). These two materials have very different properties, which might lead to significant differences in their impact on DASS. However, no such difference was found, even though none of the studies found was specifically designed to detect possible differences between these two materials [5,38].

We only found two studies that focused on specific treatment strategies for DASS. Of these, Vardizer et al. did not use a standardised questionnaire for ocular surface symptoms, nor did they conduct a quantitative analysis of ocular surface variables [35]. On the other hand, Zamorano-Martin et al. conducted a quantitative and reproducible study of the ocular surface, but their study was limited by lack of blinding, possible learning curve in use of artificial tears and eyelid wipes, and lack of use of warm compresses. They also used artificial tears that contain the preservative polyquaternium-1, which was found to be cytotoxic to ocular surface cells, and this might have been an additional bias against the effectiveness of topical lubricants for DASS [49].

As a result, more research is needed on topical lubricants for DASS.

We only found one paper on topical anti-inflammatory treatments for DASS, despite inflammation being a well-known contributor to this disease and despite a substantial body of literature on anti-inflammatory treatments such as corticosteroids and cyclosporine A in other forms of dry eye disease. It seems plausible that a dry and inflamed socket would benefit from using both topical anti-inflammatory drugs and lubricants. The only study found has encouraging results, but a controlled study on a wider population studied for a longer period would add significant evidence to support its widespread use [45].

Future research should aim to use standardised diagnostic tools, further characterise molecular biomarkers, and develop evidence-based interventions to improve comfort and quality of life for prosthetic eye wearers. Increased awareness and interdisciplinary collaboration between ophthalmologists, ocularists, and researchers are essential to advance the care of this often-overlooked condition.

## Figures and Tables

**Table 1:** Diagnostic criteria for dry anophthalmic socket syndrome (DASS).

DASS Diagnostic Criteria
Subjective symptoms (one of the following): OSDI ≥ 13 DEQ-5 ≥ 6 SANDE ≥ 13
Clinical abnormalities (one of the following): Conjunctival inflammation with staining Reduced tear meniscus height Anterior blepharitis Posterior blepharitis

Abbreviations: Ocular Surface Disease Index (OSDI), 5-item Dry Eye Questionnaire (DEQ-5), Symptom Assessment In Dry Eye (SANDE).

**Table 2:** Diagnostic tools for dry anophthalmic socket syndrome (DASS). We strongly encourage use of patient questionnaires and documentation of specific clinical signs in everyday practice. Ancillary tests might not be widely available but might provide useful additional information, and therefore should be incorporated whenever possible, especially in research.

**Patient questionnaires**
OSDI
DEQ-5
SANDE
**Clinical examination**
Meibomian gland exam and expression
Tear meniscus height
Conjunctival staining (fluorescein, rose Bengal, lissamine green)
**Ancillary tests**
Meibography
Confocal microscopy of Meibomian glands
Conjunctival impression cytology
Fourier domain optical coherence tomography of tear meniscus
Tear film osmolarity
Matrix-metalloproteinase 9 (MMP-9) immunoassay

Abbreviations: Ocular Surface Disease Index (OSDI), 5-item Dry Eye Questionnaire (DEQ-5), Symptom Assessment In Dry Eye (SANDE).

**Table 3:** Treatment recommendations.

**General measures/prevention**
Periodic prosthesis maintenance (by ocularist)
Prosthesis care (by the patient)
Control of other ocular surface conditions (e.g., allergy)
**First-line treatments**
Artificial tears and warm compresses
Punctum plugs Cyclosporine A
Surgical repair for: Lower eyelid malpositions Ptosis Surface or volume deficits

## Data Availability

The data presented in this study are available in PubMed at https://pubmed.ncbi.nlm.nih.gov. These data were derived from the following resources available in the public domain: https://pubmed.ncbi.nlm.nih.gov.
